# Genetic and molecular origins of colorectal Cancer among the Iranians: an update

**DOI:** 10.1186/s13000-018-0774-0

**Published:** 2018-12-22

**Authors:** Mohammad Reza Abbaszadegan, Meysam Moghbeli

**Affiliations:** 1 Medical Genetics Research Center, Mashhad University of Medical Sciences, Mashhad, Iran; 2 Department of Modern Sciences and Technologies, Faculty of Medicine, Mashhad University of Medical Sciences, Mashhad, Iran

**Keywords:** Colorectal Cancer, Marker, Diagnosis, Iran, Genetic

## Abstract

**Background:**

Colorectal cancer (CRC) is one the leading causes of cancer related deaths among Iranians. Despite the various progresses in new therapeutic methods, it has still a low rate of survival. This high ratio of mortality is mainly related to the late diagnosis, in which the patients refer for treatment in advanced stages of tumor.

**Main body:**

colorectal cancer progression is largely associated with molecular and genetic bases. Although Iran has a high ratio of CRC mortality, there is not an efficient genetic panel for detection and prognosis. Therefore, it is critical to introduce new diagnostic markers with ability to detect in early stages.

**Conclusion:**

Present review summarizes all of the genetic and epigenetic factors which are reported in CRC until now among the Iranian patients to pave the way of incorporation of new ethnic specific markers into the clinical practice and development of new targeted therapeutic methods.

## Background

**Fig. 1:**
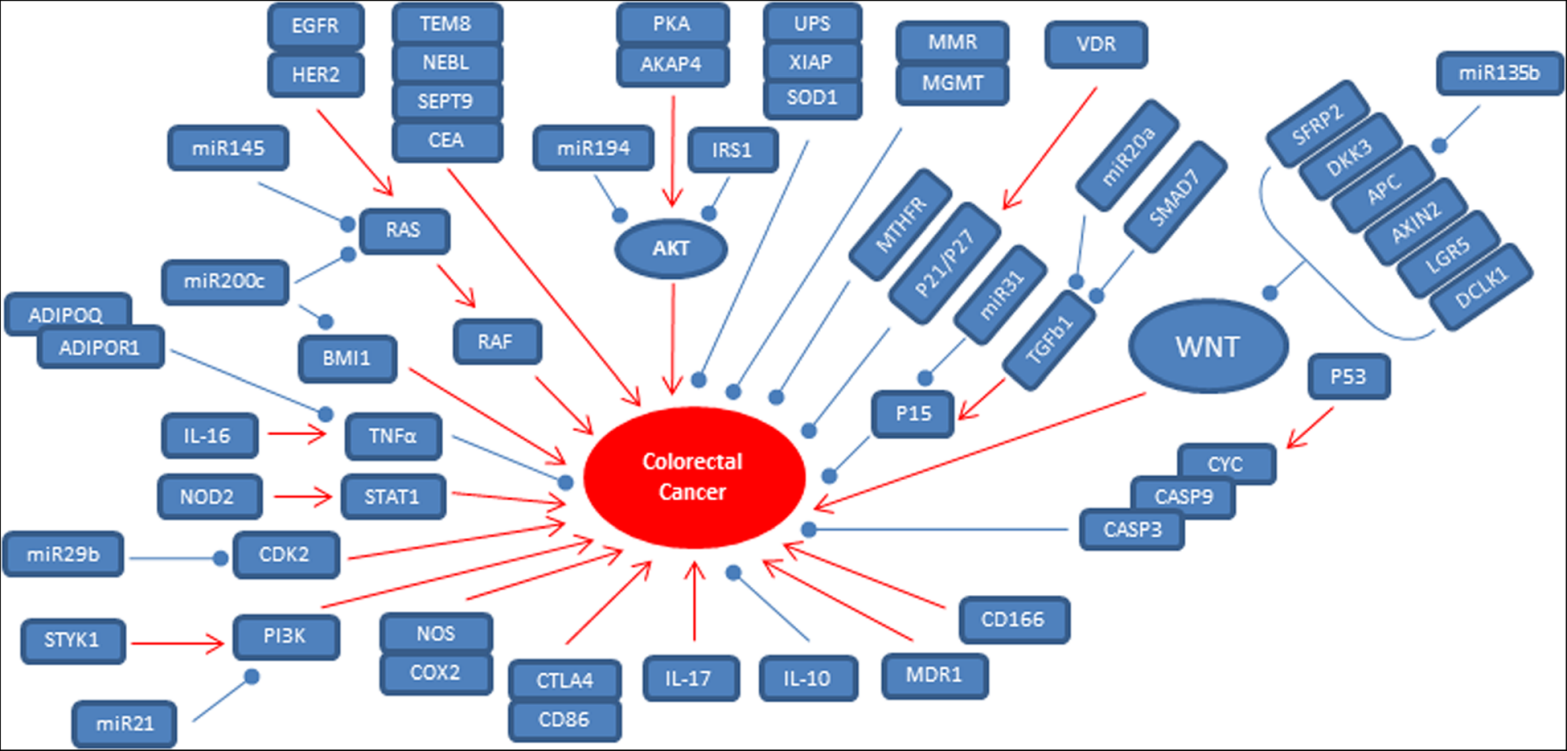
all of the interactions and signaling pathways of reported markers which are involved in CRC progression among Iranian patients.

Colorectal carcinoma (CRC) is one of the leading causes of cancer related deaths among Iranians with age-adjusted rates of 8.2 and 7.0 per 100,000 populations for men and women, respectively [1]. It was reported that there is a noticeable increase in the incidence of colorectal cancer among young Iranians [2]. Various factors are involved in individual’s susceptibility for the CRC including environmental factors and genetic predisposition [3]. Cancer is a multi-step process associated with accumulation of genetic and epigenetic alterations [4]. Gene mutations generate the genome instability via disruption of the normal epigenetic patterns [5]. Regarding the high survival ratios among the CRC cases who are diagnosed in the early stages of CRC, it is a critical requirement to find new non-invasive biomarkers to detect the CRC in the early stages [6]. Moreover, personalized medicine is also trying to design the chemotherapy based on genetic and epigenetic patterns of cancer in every patient [7]. Mutated genes are mostly oncogenes, tumor suppressor genes, and DNA repair factors which are involved in genome instability. Various processes are involved in epigenetics such as DNA methylation and histone modification. Epigenetic mutations alter the gene expression without any change in DNA sequence and can result to inactivation and activation of tumor suppressor and oncogenes, respectively. There is not any single universal molecular prognostic and diagnostic marker. Regarding the role of ethnicity on prevalence of CRC, it is believed that the different races have probably different genetic and epigenetic patterns of tumorigenesis [8]. Therefore, in present review we are going to summarize all of the 60 epigenetic and genetic aberrations which are reported until now among Iranian CRC patients (Table.1) (Fig. 1) to pave the way of designing an ethnic specific panel of diagnostic and prognostic markers in our country.

**Table 1:** All of the involved markers in CRC susceptibility among the Iranian patients

STUDY (ET AL)	Year	Gene	population	Results
Nomani [14]	2005	GST	39 controls 60 patients	Over activity in tumors
Ebrahimkhani [15]	2012	GSTT1	100 controls 100 patients	Polymorphism was correlated with CRC risk
Jamhiri [16]	2017	SOD1	239 controls 204 patients	Polymorphism was correlated with CRC risk
Mokarram [19]	2015	UBE2Q1	50 controls 60 patients	Hyper Methylation
Mokarram [19]	2015	UBE2Q2	50 controls 60 patients	Un methylation and stage of tumor
Amirghofran [22]	2009	TGFB1	138 controls 134 patients	Polymorphism was correlated with CRC risk
Damavand [24]	2015	SMAD7	253 controls 234 patients	Polymorphism was correlated with CRC risk
Naghibalhossaini [29]	2012	AXIN2	170 controls 112 patients	Methylation with stage and sex
Samaei [30]	2014	APC, AXIN2, DKK3, SFRP2, 4, 5, WIF1, and WNT5a	125 N/T^a^	Methylation
Naini [31]	2016	SFRP2	25 controls 48 polyps	Hyper Methylation as precancerous marker
Nemati [34]	2015	IL-17A	203 controls 202 patients	Polymorphism was correlated with CRC risk
Azimzadeh [38]	2011	IL-16	405 controls 260 patients	Polymorphism was correlated with CRC risk
Al-Samadi [39]	2016	IL-17F	10 tumors	Under expression in tumors
Ahangari [42]	2014	NOD2	88 controls 88 patients	Polymorphism was correlated with CRC risk
Azimzadeh [43]	2013	CD86	150 controls 150 patients	Polymorphism was correlated with CRC risk
Abtahi [44]	2017	IL-10	30 controls 58 patients	Lower levels in CRC patients
Habibollahi [49]	2010	COX2, iNOS	17 patients	Correlation with lymph node metastasis
Hadinia [52]	2007	CTLA4	190 controls 109 patients	Polymorphism was correlated with CRC risk
Shafaei [53]	2013	CD166	121 N/T	Correlation with tumor location
Mahmoudi [56]	2016	ADIPOQ, ADIPOR1	339 controls 261 patients	Polymorphism was correlated with CRC risk
Motlagh [58]	2007	EGFR	34 N/T	Marker of preoperative therapy response
Tavangar [59]	2005	HER2	55 N/T	Correlation with tumor size, stage, differentiation
Orang [61]	2014	STYK1/NOK	40 N/T	Correlation with tumor size, stage
Tavakoli Koudehi [63]	2018	AKAP4	62 N/T	Correlation with liver metastasis in CRC patients
Hamzehzadeh [65]	2018	KRAS	87 patients	Mutation
Koochak [68]	2016	KRAS	1000 patients	Correlation with age, tumor type
Omidifar [69]	2015	KRAS	100 patients	Mutation
Ghavam-Nasiri [71]	2007	P53	100 patients	Correlation with stage
Asadi [74]	2018	CASPASE3	50 N/T	Under expression
Golmohammadi [75]	2013	P53	61 patients	Correlation with distal tumors, sex, survival
Moossavi [77]	2018	VDR	100 controls 100 patients	Polymorphism was correlated with CRC risk
Mahmoudi [78]	2010	VDR	180 controls 160 patients	Polymorphism was correlated with CRC risk
Mahmoudi [82]	2016	IRS1	438 controls 312 patients	Polymorphism was correlated with CRC risk
Karimi Mazraehshah [86]	2018	miR-200c, BMI1	38 controls 38 patients	Under an over expression, respectively
Orang [90]	2014	miR-205	36 N/T	Correlation with lymph node metastasis, stage
Basati [91]	2014	miR-21	40 controls 40 patients	Correlation with stage
Bastaminejad [92]	2017	miR-21	40 controls 40 patients	Correlation with stage
Basati [95]	2016	miR-194	55 controls 55 patients	Correlation with stage
Eslamizadeh [105]	2018	miR-135b, 20a, 31, 21	32 controls 74 patients	Increased levels in CRC patients
Montazer Haghighi [107]	2009	MMR	248 controls 592 patients	Novel mutations in PMS2 and MLH1
Shahmoradi [108]	2012	hMLH1	20 patients	Novel mutations
Moghbeli [110]	2011	MMR	67 N/T	MSI was correlated with location, stage, lymph node metastasis
Faghani [111]	2012	MMR	96 N/T	MSI was correlated with location
Nazemalhosseini Mojarad [112]	2016	MMR	158 N/T	MSI was correlated with differentiation, stage, sex
Brim [113]	2008	MMR	53 patients	MSI was correlated with differentiation
Khatami [114]	2009	MGMT	213 controls 208 patients	Polymorphism was correlated with CRC risk
Farzanehfar [115]	2013	MGMT	40 N/T	Methylation
Nemati [117]	2018	HDAC3	48 N/T	Correlation with stage, differentiation
Ayoubi [121]	2017	H3.3B	36 N/T	Correlation with stage, lymph node
Mohammed [125]	2015	tRNA^Leu(CUN)^	100 controls 30 patients	Mutation
Haghighi [128]	2008	MTHFR	239 controls 227 patients	Polymorphism was correlated with CRC risk
Naghibalhossaini [131]	2010	MTHFR	231 controls 175 patients	Polymorphism was correlated with CRC risk
Samanian [136]	2011	MDR1	60 controls 60 patients	Correlation with grade
Khedri [137]	2011	MDR1	137 controls 118 patients	Correlation with stage
Ardalan Khales [138]	2015	SALL4	111 N/T	Correlation with grade, depth of invasion
Raeisossadati [143]	2014	DPPA2, HIWI	46 N/T	Correlation with stage, lymph node metastasis
Mirzaei [146]	2015	LGR5, DCLK1	58 controls 58 patients	Correlation with stage
Niknami [149]	2017	FIBRONECTIN	45 patients	Correlation with grade
Hosseini [150]	2018	NEBL	67 N/T	Correlation with lymph node metastasis
Behrouz Sharif [153]	2016	SEPT9	45 N/T	Methylation
Raeisossadati [154]	2011	CEA, TEM8	40 controls 40 patients	Over expression in CRC patients

^a^Tumor tissues and normal margins

## Main text

### Intracellular defense systems

Glutathione S-transferases (GSTs) are involved in detoxification of carcinogens and are expressed in many tissues such as gastrointestinal tracts [9]. GSTs are belonged to the phase II metabolic enzymes which have critical roles in cellular detoxification. Phase I (activation) and phase II (conjugation) enzymes are known as the most important factors involved in metabolization of carcinogens [10, 11]. GSTs over activity was observed in chemotherapeutic resistant tumors against the alkylating agents [12, 13]. GST activity in plasma and tissue were assessed in Iranian CRC cases. There was a significant GSTs over activity in tumor compared with normal margins. This strong correlation supported the plasma GST activity as a non-invasive biomarker [14]. GSTM1, P1, T1, and CYP2E1 polymorphisms were also assessed among Iranian CRC subjects. They reported that there was a strong correlation between GSTT1 polymorphism and CRC [15]. Superoxide dismutase (SOD) and catalase are the main protective enzymes against reactive oxygen species. SODs convert the superoxide into hydrogen peroxide, subsequently the catalase is responsible for the hydrogen peroxidase detoxification through converting that into the water and oxygen. Therefore, a group have assessed the correlation between SOD1 A251G and CAT C-262 T polymorphisms and CRC susceptibility among the Iranian population. They observed that the SOD1 G allele was correlated with a higher risk of CRC. Therefore, polymorphisms in antioxidant enzymes increase risk of CRC through the decreased antioxidant capacity [16]. Beside the toxic compounds and ROS, abnormal and defective proteins can be also damaging for the cells, therefore it will be required for the cells to eliminate such abnormal proteins. Ubiquitin-proteasome system (UPS) regulates various cellular processes such as cell differentiation, proliferation, and apoptosis [17]. It was reported that deregulation of ubiquitin-proteasome can be involved in tumor progression through the modulation of growth factors stability. UBE2Q1, 2 are belonged to the E2 ubiquitin-conjugating enzyme family [18]. Methylation status of UBE2Q1 and UBE2Q2 were assessed in a group of Iranian CRC cases. UBE2Q2 was highly un-methylated in distal and advanced stage tumors suggesting the UBE2Q2 as a probable oncogene during the CRC progression. It was observed that the UBE2Q1 hypermethylation and UBE2Q2 hypomethylation as tumor suppressor and oncogene respectively can be associated with CRC progression [19].

### TGF-b and WNT signaling pathways

Transforming growth factor b (TGF-b) act as a tumor suppressor via cell proliferation arrest and apoptosis induction during early stages of tumor progression [20]. TGF-b1 binding with receptor type II (TGF-bRII), results to phosphorylation of the receptor type I (TGF-bRI). This interactions finally activates and transfers the SMADs into the nucleus, resulting in the transcriptional regulation of target genes which are related to the cell proliferation [21]. Role of TGFB1 gene − 509 C/T polymorphism in risk of CRC was evaluated in a group of Iranian patients. They showed that the cases with the TGFB1 509 T allele can be at a lower risk of CRC in comparison with the cases harboring 509 C allele. This study highlighted role of this cytokine in suppression of CRC in which the 509 T allele has probably a protective role during the tumor progression [22]. SMAD7 is an inhibitor of the TGF-β which functions as an oncogene through the suppression of TGF-β signaling [23]. It was shown that there was a significant difference between the rs4464148 AG frequency of SMAD7 in the CRC and control groups. It was demonstrated that the allele T at rs12953717 of SMAD7 can be introduced as a risk factor of CRC among Iranian patients [24]. Canonical Wnt/b-catenin is one of the critical pathways during the tumorigenesis in which the unphosphorylated and activated b-catenin enters into nucleus and activates the LEF/TCF/PYGO2 transcriptional complex [25]. GSK3b, AXIN, and APC are the components of a complex which is responsible for the regulation of b-catenin. This complex phosphorylates and induces the degradation of b-catenin via the proteasome in the absence of WNT signals [26]. Regarding the presence of APC mutation in more than 80% of CRCs, aberrant WNT signaling pathway has a critical role in CRC progression [27, 28]. It was observed that the 7 and 12% of a group of 112 Iranian CRC patients had AXIN2 and APC promoter methylation. AXIN2 promoter methylation was mainly observed in stage I tumors and females [29]. Another group also examined the promoter methylation status of several WNT inhibitors among 125 Iranian CRC patients. They observed the APC, AXIN2, DKK3, SFRP2, 4, 5, WIF1, and WNT5a promoter methylation in 35.2, 32.8, 40, 46.4, 28.8, 26.4, 41.6, 22.4% of tumor samples, respectively [30]. It was reported that the hypermethylation of APC1A and SFRP2 promoter sequences as the WNT target genes are also correlated with polyp progression. Therefore, hypermethylation of serum SFRP2 can be suggested as a sensitive precancerous marker [31].

### Inflammatory factors

The cell microenvironment and tissue homeostasis and repair can be modulated by inflammatory responses [32]. Adaptive and innate immune systems have important roles in cancer. IL-17 is an inflammatory cytokine secreted by Th17 cells and stimulates the Macrophages and Neutrophils to produce the inflammatory factors in human malignancies [33]. Frequencies of IL-17 SNPs were assessed in CRC tumors and serum levels of IL-17 were compared in patients and healthy controls. It was found that the TT genotype and T allele were decreased in comparison with the TC genotype and C allele among Iranian CRC patients. There was a significant correlation between IL-17F TT genotype and well differentiation. There was also a significant correlation between the AG genotype of IL-17A G197A SNP and higher risk of colorectal cancer. In contrast with the IL-17F, IL-17A serum concentrations were significantly increased in CRC compared with healthy cases. Therefore, IL-17A is a tumor progression cytokine and can be efficiently introduced in targeted therapy of CRC patients [34]. IL-16 is a chronic inflammation cytokine mediating tissue specific or systemic inflammation [35, 36]. It was observed that the IL-16 up regulates the TNF-α as an important factor in apoptosis and cell survival [37]. Single nucleotide polymorphisms in promoter and exons of IL-16 were screened to assess the frequency of genotypes in Iranian CRC and healthy cases. The results suggested a correlation between rs11556218 T > G and rs4778889 T/C polymorphisms and risk of CRC among a sub population of Iranian patients [38]. Expression of IL-17B, C, E, and F were also examined among Iranian sporadic CRC cases. Although the IL-17B was increased, IL-17C showed a grade-linked expression pattern and IL-17E remained unchanged in CRC patients. IL-17F under expression was observed in tumors in comparison with the normal samples [39]. NOD2 protein is a regulator of chronic inflammatory that is expressed in monocytes and macrophages. It functions as a sensor of lipopolysaccharides and peptidoglycans to induce the (NF)-jB and STAT1 transcription factors which are associated with inflammation-related tumor progression [40, 41]. Correlation between rs3135500 (30UTR SNP) of NOD2 and CRC susceptibility was evaluated among the Iranian population. It seems that the rs3135500 SNP is involved in deregulation of NOD2 through changing the mRNA–miRNA interaction. There was a significant correlation between AA genotype of rs3135500 and increased risk of CRC in Iranian population [42]. CD86 is expressed by immune cells and is associated with inflammation related malignancies and cancer susceptibility. The 3’UTR + 237 G/C polymorphism of CD86 was assessed among 300 Iranian CRC cases and showed a strong correlation between rs17281995 polymorphism and risk of CRC [43]. IL-10 as an anti-inflammatory cytokine is commonly secreted by type 1 regulatory T cells and can be an oncogene or tumor suppressor based on microenvironment condition. A case–control study showed that there was significantly lower serum levels of IL-10 in Iranian CRC cases compared with controls which cause an aberrant innate immune reaction and tumor cell ignorance by adaptive immune. Moreover, the cases with poor prognosis had higher levels of IL-10 compared with the other cases [44]. Nitric oxide (NO) is involved in DNA damage and apoptosis inhibition [45, 46]. Its production is related with three isoforms of nitric oxide synthase (NOS) including endothelial (eNOS), neuronal (nNOS), and inducible (iNOS). eNOS and nNOS activations are calcium dependent [47], whereas, iNOS is related to the cytokines and has a calcium independent function. It has been shown that the NO is involved in prostaglandin biosynthesis through the COX-2 activation during inflammation process [48]. It was shown that the COX2 and iNOS protein co-expressions were observed in epithelial tumor cells. The cases with lymph node metastasis showed higher levels of COX2 and iNOS protein expressions. Therefore, there was a direct correlation between iNOS and COX2 in advanced stage tumors among the Iranian CRC subjects [49]. Cytotoxic T-lymphocyte antigen-4 (CTLA-4) is one of the critical factors in immune responses against different antigens and is expressed by activated T cells [50, 51]. It was shown that the TACG haplotype of CTLA-4 gene can be associated with high susceptibility to CRC among Iranian population [52]. Role of CD166 was studied in Iranian CRC cases using an immunohistochemical method, and it was shown that 71.1, 34.7, and 34.7% of cases were positive in cytoplasmic, membranous, and simultaneous expressions, respectively. Non-mucinous types had higher levels of cytoplasmic CD166 expression. Moreover, there was an association between tumor location and CD166 expression in which the tumors in right colon were related with CD166 membranous expression [53]. Adiponectin is mainly produced by adipose tissue and has anti-inflammatory and anti-proliferative functions [54, 55]. ADIPOQ (rs2241766) and ADIPOR1 (rs2275738) gene variants were assessed among Iranian CRC cases. It was reported that there was a correlation between ADIPOQ rs2241766 “G” allele and decreased risk of CRC among obese patients. Moreover, the cases with ADIPOR1 rs2275738 “CC + CT” had about 49% higher risk of CRC in comparison with the “TT” genotype [56].

### Kinases and growth factors

EGFR is a tyrosine kinase and transmembrane glycoprotein which is dimerized following the activation and exerts an intrinsic tyrosine kinase activity. Subsequently, this factor triggers a cytoplasmic cascade toward the cell proliferation and differentiation [57]. It was observed that the expression rates was 41% among a group of Iranian CRC patients with pathologic response of 59%. They indicated that EGFR expression can be used as an important marker of pathologic response to preoperative treatment in which the EGFR expression was related with a lack of pathologic response [58]. Another group also reported a HER2 over expression in 21.8% of Iranian colorectal cancer patients and there was significant correlations between HER2 over expression and tumor size and advanced stages. Moreover, the HER2 over expression was more frequent in moderate and poorly differentiated tumors [59]. STYK1/ NOK is a tyrosine kinase receptor that can be activated in a non-specific status due to lacks extracellular domain [60]. Levels of STYK1/NOK expression were evaluated in a group of Iranian CRC cases. It was shown that there was a noticeable STYK1/NOK over expression in CRC tissues and a significant correlation between STYK1/NOK over expression and tumor size. They observed the high levels of STYK1/NOK expression in the early clinical stages of CRC [61]. Although, Cancer-Testis antigens (CTAs) are expressed in normal testis, their aberrant expression is observed in different tumors [62]. AKAP4 is one of the members of CTA family which binds to protein kinase A (PKA) to connect that to the specific cellular locations. Levels of AKAP4 expression were assessed in Iranian CRC patients which showed a significant correlation between AKAP4 expression and liver metastasis in CRC cases. AKAP4 was expressed in normal testis and CRC tissues but not in normal margins, introducing that as a diagnostic CRC marker [63]. Between 30 and 40% of CRC tumors harbor the mutated KRAS gene [64]. The N-Ras is a guanosine triphosphatase membrane protein which is observed mutated in 1–6% of CRC cases. Frequency of KRAS mutations was assessed in 87 Iranian CRC patients. They showed a 28.7% of KRAS mutation that was in the range of other Asian and western countries. They showed a similarity in the KRAS hotspot mutations between Iranian and other countries CRC patients [65]. The KRAS activates BRAF through a signal transduction cascade including EGFR and MAPK [66, 67]. Mutational analysis of KRAS and BRAF genes were performed in a group of Iranians with metastatic CRC using Pyrosequencing method. KRAS mutation was observed in 33.6% of CRC cases, which involves mutations at codon 12 and 13. There was a significant correlation between KRAS mutations and older ages and tumor type (non-mucinous) [68]. Incidence of mutation in codons 12 and 13 of KRAS was screened by another group among Iranian CRC subjects, showed that the codon 13 mutations had lower frequencies compared with codon 12 (8% vs. 24%). Moreover, up to 32% of cases harbors mutations in both of codon 12 or 13 [69].

### Apoptosis

The p53 tumor-suppressor gene has an important role in cell cycle regulation following the DNA damage. Moreover, p53 as a transcription factor is involved in regulation of more than 100 different factors [70]. It stimulates transcription of factors involving in the cell cycle arrest and apoptosis. Levels of p53 protein expression was assessed in a group of Iranian CRC cases. They observed higher percentage of p53-positive expression in advanced stages, introducing that as a genetic marker for tumor relapse prediction and response to chemotherapy in Iranian CRC cases [71]. Caspase 8 is one of the components of extrinsic apoptosis pathway. Although the intrinsic pathway is related to the cytochrome c release from mitochondria and caspase 9 activation, both of intrinsic and extrinsic pathways are merged in activation of caspase 3 [72, 73]. It has been shown that there was a caspase 3 under expression in tumor compared with normal CRC samples. Moreover, aberrant caspase 8 and 9 mRNA expressions were also observed in CRC in comparison with normal margins [74]. Mutational analysis of exons 5 and 6 in p53 were also studied in a subpopulation of Iranian CRC patients. Twenty one point mutations including two novels were observed. There was an association between frequency of mutations and distal tumors. Moreover, females had lower mutation rates in comparison with males (13% vs 26%), and the cases with mutated p53 had shorter survival in comparison with the none mutated cases [75].

### Vitamin D and insulin

It has been reported that the vitamin D interaction with its receptor (VDR) is associated with CRC susceptibility [76]. It was observed that the ff (TT) genotype of rs2228570 can be suggested as a risk factor genotype for CRC susceptibility among a group of Iranian CRC patients [77]. Moreover, another group also reported that the VDR ApaI genotype “aa” is correlated with higher risk of CRC among a sub population of Iranian patients [78]. Insulin is involved in cell proliferation and has an anti-apoptotic role in the target tissues. Hyperinsulinemia has been proposed as an important process to link the obesity with CRC [79]. Type 2 diabetes mellitus in association with insulin resistance and obesity increase the CRC susceptibility [80]. Insulin receptor substrate 1(IRS1) has a key function in cell proliferation [81]. Association of IRS1 (rs1801278) with CRC risk was assessed and showed that the IRS1 G972R R allele and RR + GR genotype have protective roles in obese Iranian CRC patients [82].

### MicroRNAs

MicroRNAs are noncoding RNAs that regulate several cellular processes such as proliferation and migration. They exert their regulatory function through the mRNA degradation or translational suppression via 3′-untranslated regions [83]. MiR-200c inhibits the cell proliferation and EMT and stimulates the chemo sensitivity and apoptosis [84, 85]. BMI1 as a components of polycomb repressive complex is involved in chromatin remodeling, cell cycle regulation, DNA repair, and self-renewal. Expression of miR-200c and BMI1 were assessed in a group of Iranian CRC cases, showing an under and over expression of miR-200c and BMI1 respectively in tumors compared with normal margins. Therefore, there was a converse correlation between miR-200c and BMI1 in which the miR-200c exerts its tumor suppressor role via BMI1 inhibition in CRC patients [86]. MiR-205 is an epithelial-specific miRNA involving in EMT and cell differentiation [87]. It functions as a tumor suppressor or oncogene depending on the targeted mRNA [88, 89]. It was observed that there was a correlation between the lower levels of miR-205 expression and lymph node metastasis. Therefore, it seems that the loss of miR-205 can be involved in advanced stages of CRC progression in which de-differentiated tumor cells migrates via an EMT process [90]. MiR-21 as an oncogene targets several important factors such as PTEN, PI3K, BTG2, FasL, and FBXO11. Serum level of miR-21 was analyzed and showed a significant elevated levels in CRC in comparison with control cases. Moreover, there was a significant correlation between serum miR-21 levels and tumor stage, suggesting this marker as an efficient prognostic factor among Iranian CRC subjects [91]. Another group also reported the elevated levels of miR-21 in stool and serum of Iranian CRC patients. It was shown that the levels of miR-21 expression in tumor cells were associated with stool and serum. There was also a significant correlation between miR-21 over expression and tumor stages of I and II. Therefore, miR-21 expression in serum and stool can be introduced as an efficient detection marker among the Iranian CRC patients [92]. MiR-29b targets several important factors such as MMP-2 and CDK2 which are important for the epithelial cell growth [93, 94]. Another group have also assessed the serum levels of miR-194 and miR-29b and observed a significant decrease in CRC compared with the control subjects. There were correlations between declined serum miR-194 and miR-29b levels and poor prognosis and tumor stage suggesting the miR-194 as a tumor suppressor in CRC cases. Therefore, serum miR-194 and miR-29b levels can be used as a non-invasive prognostic and diagnostic method among the Iranian CRC patients [95]. MiR-200c prohibits the EMT through downregulation and upregulation of ZEB1/2 and E-cadherins, respectively [96, 97]. MiR-145 exerts its tumor suppressor role via inhibition of various factors such as MYC, Kras, and SOX2 [98, 99]. MiR-135b as an oncogene also targets different tumor suppressors such as LATS1–2 and APC [100]. HIF-1α and CDKN2B tumor suppressors are the targets of miR-31 [101, 102]. MiR-20a is involved in tumor progression through down regulation of several factors including Smad4, E-cadherin, and TGF-β [103, 104]. Expression of eight miRNAs including miR-31, miR-135b, miR-133, miR-21, and miR-20a were also examined in tumor and plasma of CRC cases. The levels of miR-21, miR-31, miR-20a and miR-135b were significantly increased in both plasma and tissue of CRC cases in comparison with the control group. There were significant differences between the levels of miR-21 and miR-20a expressions and stage of tumor, in which the plasma levels of miR-21 and miR-20a showed a significant rising from the early to advanced stages of CRC. Therefore, they introduced the miR-135b, 20a, 31 and 21 as probable efficient diagnostic factors among Iranian CRC patients [105].

### DNA repair and microsatellite instability

Accumulation of genetic abnormalities is one of the main reason of CRC progression. About 85% of CRCs initiate from chromosomal instability, whereas the others are associated with microsatellite instability (MSI). MSI is due to the failure of mismatch repair (MMR) system leading to aberrant replication [106]. Assessment of MMR germline mutations among the Iranian HNPCC cases showed four novel mutations in PMS2 and MLH1 [107]. Mutational analysis of hMLH1 was also performed in a sub population of Iranian HNPCC, and showed two novel frame shift mutations at exons 1 and 19. They suggested that the determination of hMLH1 regional mutation patterns can be helpful to design efficient preventive strategies to decrease the HNPCC mortality [108]. Standard microsatellite NCI/ICG panel [109] was assessed in sporadic CRC patients in the northeastern Iran. MSI was observed in 43.3% of our patients and 26.9% of cases had MSI-H phenotype. MSI was correlated with right-sided and lower stage tumors whereas MSS was correlated with lymph node metastasis. MSI testing was suggested to determine effective chemotherapeutic regimen in our population [110]. Another study also used BAT-25 and BAT-26 markers to assess the correlation between MSI and sporadic CRC among the cases from northern Iran and showed a similar incidence of these markers. They showed that there was an association between tumor location and MSI-H in which 81.8% of MSI-H tumors were located in distal colon. Moreover, among MSI-H cases the distal tumors had higher rate of lymph node metastasis compared with proximal tumors (74.2% vs. 25.8%). Generally, metastasis ratio in MSI-H was higher than that in MSS tumors in right and left tumors [111]. The correlation between prognosis and MSI was assessed in CRC patients and showed that the MSI-H tumors were significantly correlated with poorer differentiation and stage II/III of tumors. There was also a significant correlation between MSI status and gender in which the males had a high incidence of MSI-H. In the case of stage II samples, MSI-L cases had significantly poorer survival rate in comparison with the MSI-H cases [112]. Microsatellite instability and BRAF mutational analysis in Iranian CRC population have shown 13% of instability and 2% of BRAF mutation, respectively. MSI was more frequent in proximal tumors and MSI-H was correlated with higher tumor differentiation [113]. The O6-methylguanine DNA methyltransferase (MGMT) maintains genomic stability via alkylated DNA repair. Frequency of polymorphism in MGMT and DNMT1 were assessed in a group of Iranian CRC patients and there was significant correlations between Arg128Gln and Gly160Arg polymorphisms in MGMT and risk of CRC [114]. In another report, the MGMT promoter methylation was also evaluated using a QMSP method in a sub population of Iranian CRC cases. It was observed that the tumor tissues had significantly higher levels of MGMT methylation compared with control tissues. Therefore epigenetic inactivation of MGMT through promoter hypermethylation can be introduced as an efficient method of early detection among the Iranian CRC patients [115].

### Chromatin remodeling factors

Aberrant DNA methylation and histone modifications play important roles during tumor progression. Histone acetyltransferases (HATs) induce the chromatin access and gene transcription; while, histone deacetylases (HDACs) condense the chromatin and inactivate transcription [116]. Levels of HDAC3 gene expression were assessed in a group of Iranian CRC cases showing a high expression in the tumors. There was correlation between HDAC3 over expression and advanced stages of disease and poorly differentiation. Therefore, HDAC3 can be introduced as a prognostic biomarker and efficient therapeutic target among the Iranian CRC patients [117]. Histone family including H1, H2A, H2B, H3, and H4 are the most important factors which are responsible for the chromatin condensation. H3.3 histone is encoded by H3.3A and B and overexpressed in promoter sequences and transcriptionally active regions [118, 119]. EGFR activates the MAPK signaling pathway which induces the transcription factors such as CREB and ATF1 to regulate the H3.3B [118, 120]. Then, H3.3 histone converts the heterochromatin to euchromatin and activates the factors which are involved in tumor progression [118, 119]. H3.3B over expression was observed in tumor compared with normal margins among a sub population of Iranian CRC subjects. There was significant correlations between levels of H3.3 expression and tumor stage III and lymph node metastasis. Therefore, H3.3B can be probably introduced as a prognostic marker in CRC [121].

### Mitochondrial DNA

Mitochondria are the only organelles in eukaryotes with extra chromosomal DNA. Human mitochondria encode polypeptides and ribonucleic acids which are involved in oxidative phosphorylation. Moreover, there is a D-loop non coding region involving in regulation of replication and transcription in mitochondrial DNA. Therefore, D-loop mutations can be resulted to the accumulation of reactive oxygen species and DNA damage and tumorigenesis [122]. D-loop mutations have been screened in a group of Iranian CRC patients compared with healthy cases. They observed that there was higher frequencies of SNP in CRC patients in comparison with control group. Frequencies of eight SNPs were shown to be significantly different between CRC and control groups, introducing these SNPs as probable risk factors among CRC patient. There was also one thymine to cytosine transition at np16519 in 70% of CRC cases [123]. Majority of mtDNA mutations have been observed in mt-tRNA genes [124]. The human mitochondrial A12308G mutation in tRNA Leu (CUN) was assessed in a sub population of Iranian CRC patients. They observed a significant higher frequency of A12308G polymorphic mutation in V-loop (tRNA^Leu(CUN)^) among the CRC patients compared with healthy controls [125].

### Methylation and DNA synthesis

Folate as the main source of methyl groups is involved in DNA repair, methylation, and synthesis. Methylenetetrahydrofolate reductase (MTHFR) is a key enzyme in folate metabolism and methionine production which is a methyl donor for DNA [126]. Therefore folate deficiency may results to the oncogenic DNA hypomethylation [127]. Correlation between the SNP in MTHFR and risk of CRC was assessed in a group of Iranian population, showing a converse correlation between codon G1793A and colon cancer. Their results showed an inverse correlation between MTHFR1793 genotype with colorectal cancer among Iranians [128]. Folate aberration may lead in higher DNA replication errors and chromosomal breaks via misincorporation of uracil into DNA [129, 130]. Therefore, it has been shown that MTHFR aberration can be associated with MSI in CRC. A case–control study has been evaluated the role of C677T and A1298C polymorphisms in tumor susceptibility and MSI in a group of Iranian CRC patients. They observed that the MTHFR 677 T allele was strongly correlated with CRC and MSI which can be due to MMR inactivation through promoter hypermethylation [131].

### Drug resistance

Chemotherapeutic treatments are the most efficient therapeutic methods, however drug resistance in many tumors is a big problem in cancer therapy [132, 133]. Multi drug resistance (MDR) is a process of drug resistance in tumor cells against a wide range of drugs [134]. The multi-drug resistance (MDR1) is belonged to the ABC transporters with ability of drug exclusion from the cells through an energy-dependent efflux pump activity [135]. Role of several polymorphisms in expression levels of MDR1 was evaluated among a group of Iranian CRC patients. There was a significant difference between frequency of G2677 T/A polymorphism in control and patients groups. It was shown that the GG2677 and AT 2677 genotypes were correlated with highest and lowest levels of MDR1 expression, respectively. Moreover, there was a correlation between G2677 T/A polymorphism and grade of CRC tumors [136]. Another group has also reported that there was a correlation between MDR1 polymorphism and risk of CRC among Iranian patients. They reported that the 3435TT genotype plays an important role in the early steps of CRC progression [137].

### Self-renewal and Cancer stem cells

SALL4 is a zinc-finger transcription factor involving in self-renewal of stem cells. Levels of SALL4 mRNA expression have been evaluated in peripheral blood and serum of CRC cases. They showed a significant higher SALL4 copy numbers in blood and serum of CRC patients compared with normal controls. Moreover, there was significant correlations between SALL4 expression and tumor differentiation and depth of invasion. SALL4 blood copy numbers were conversely correlated with tumor depth of invasion, cases without tumor invasion into the adventitia had significantly higher levels of SALL4 copy numbers compared with patients with tumor invasion. Therefore, SALL4 can be introduced as an efficient diagnostic marker for the early detection of Iranian CRC cases [138]. DPPA2 and HIWI are also developmental factors which are associated with reprogramming and self-renewal state of stem cells [139, 140]. DPPA2 expression is limited to the germ and pluripotent stem cells. The HIWI protein has critical roles in self-renewal and proliferation of stem cells through the regulation of several processes such as protein synthesis, RNAi mechanism, and mRNA stability [141, 142]. We have shown the HIWI and DPPA2 mRNA over expressions in 34.8 and 26.1% of 46 Iranian CRC cases, respectively. HIWI and DPPA2 over expressions had significant correlations with advanced stages and lymph node metastasis, respectively. Moreover, there was a significant direct correlation between HIWI and DPPA2 mRNA expression in which DPPA2 probably regulates the HIWI expression in CRC [143]. Cancer stem cells (CSCs) are a sub population of tumor cells which are responsible for the drug resistance and tumor relapse [144, 145]. Therefore, targeted therapy against the CSCs can be an efficient method to overcome the tumor relapse. To evaluate the presence of CSCs in peripheral blood (PB), LGR5 and DCLK1 expressions have been assessed in blood sample of CRC patients. Levels of Lgr5 and DCLK1 in PB of CRC were higher than them in control group. Moreover, there was a significant correlation between Lgr5 and DCLK1 over expressions and advanced stages of CRC. Pre-operation chemoradiotherapy was also associated with higher levels of DCLK1 mRNA expression. Generally, they suggested the Lgr5 and DCLK1 as CSC markers among a sub population of Iranian CRC patients [146].

### Cell adhesion and cytoskeletal factors

The epithelial–mesenchymal transition (*EMT*) is maintained through up and down-regulation of mesenchymal and adhesion factors [147]. Fibronectin is a member of extracellular matrix and is involved in cell migration, embryogenesis, and EMT [148]. It has been observed that the levels of Fibronectin mRNA expression was increased and decreased in primary and advanced tumor grades respectively in a subpopulation of Iranian CRC patients [149]. Nebulin (NEBL) is also involved in cell adhesion through interaction with thin filaments, vinculin, and paxillin which probably increase the cell migration via destabilization of focal adhesions. Levels of NEBL mRNA expression was evaluated in CRC tumors and their corresponding normal margins among Iranian CRC cases. It was shown that the tumor tissues had three fold increases of NEBL mRNA expression in comparison with the normal tissues. There was also a significant direct correlation between levels of NEBL mRNA expression and lymph node metastasis. Therefore, they suggested that the NEBL can be introduced as a prognostic factor among the Iranian CRC patients [150]. Septin 9 (SEPT9) is a GTP-binding protein which is involved in cytoskeletal filamentous structures [151, 152]. Promoter methylation status of SEPT9 was examined using MS-HRM assay in CRC patients. SEPT9 had significant high levels of promoter methylation in tumor compared with normal margins. They concluded that the SEPT9 methylation can be introduced as a diagnostic marker among the Iranian CRC patients [153]. Carcinoembryonic antigen (CEA) as a hemophilic cell adhesion protein has important role in embryogenesis and tumorigenesis. Tumor epithelial marker 8 (TEM-8) is also involved in cell interaction with extracellular matrix. We have shown that the mRNA levels of TEM-8 and CEA in peripheral blood of CRC patients were significantly higher than that in the control group. Therefore, CEA and TEM-8 mRNA copy numbers can be introduced as markers of tumor progression among the Iranian CRC patients [154].

## Conclusion

It has been shown that the ethnic is an important issue in genetic abnormalities that are involved in tumor progression. For the first time, present review summarizes all of the genetic and epigenetic factors which are reported in CRC until now among the Iranian patients to pave the way of incorporation of new ethnic specific markers into the clinical practice and development of new targeted therapeutic options.

## Abbreviations

CEA: Carcinoembryonic antigen
CRC: Colorectal carcinoma
CSCs: Cancer stem cells
CTAs: Cancer-Testis antigens
CTLA-4: Cytotoxic T-lymphocyte antigen-4
*EMT*: Epithelial–mesenchymal transition
GSTs: Glutathione S-transferases
HATs: Histone acetyltransferases
HDACs: Histone deacetylases
IRS1: Insulin receptor substrate 1
MDR: Multi drug resistance
MGMT: O6-methylguanine DNA methyltransferase
MMR: Mismatch repair
MTHFR: Methylenetetrahydrofolate reductase
NEBL: Nebulin
NO: Nitric oxide
NOS: Nitric oxide synthase
PB: Peripheral blood
PKA: Protein kinase A
SEPT9: Septin 9
TEM-8: Tumor epithelial marker 8
TGF-b: Transforming growth factor b
UPS: Ubiquitin-proteasome system
